# Luteolin-Enriched Artichoke Leaf Extract Alleviates the Metabolic Syndrome in Mice with High-Fat Diet-Induced Obesity

**DOI:** 10.3390/nu10080979

**Published:** 2018-07-27

**Authors:** Eun-Young Kwon, So Young Kim, Myung-Sook Choi

**Affiliations:** 1 Department of Food Science and Nutrition, Kyungpook National University, 1370 San-Kyuk Dong Puk-Ku, Daegu 41566, Korea; savage20@naver.com (E.-Y.K.); ksd1372@hanmail.net (S.Y.K.); 2 Center for Food and Nutritional Genomics Research, Kyungpook National University, 1370 San-Kyuk Dong Puk-Ku, Daegu 41566, Korea

**Keywords:** artichoke, hepatic steatosis, inflammation, insulin resistance, luteolin, obesity

## Abstract

This current study aimed to elucidate the effects and possible underlying mechanisms of long-term supplementation with dietary luteolin (LU)-enriched artichoke leaf (AR) in high-fat diet (HFD)-induced obesity and its complications (e.g., dyslipidemia, insulin resistance, and non-alcoholic fatty liver disease) in C57BL/6N mice. The mice were fed a normal diet, an HFD, or an HFD plus AR or LU for 16 weeks. In the HFD-fed mice, AR decreased the adiposity and dyslipidemia by decreasing lipogenesis while increasing fatty acid oxidation, which contributed to better hepatic steatosis. LU also prevented adiposity and hepatic steatosis by suppressing lipogenesis while increasing biliary sterol excretion. Moreover, AR and LU prevented insulin sensitivity by decreasing the level of plasma gastric inhibitory polypeptide and activity of hepatic glucogenic enzymes, which may be linked to the lowering of inflammation as evidenced by the reduced plasma interleukin (IL)-6, IL-1β, and plasminogen activator inhibitor-1 levels. Although the anti-metabolic syndrome effects of AR and LU were similar, the anti-adiposity and anti-dyslipidemic effects of AR were more pronounced. These results in mice with diet-induced obesity suggest that long-term supplementation with AR can prevent adiposity and related metabolic disorders such as dyslipidemia, hepatic steatosis, insulin resistance, and inflammation.

## 1. Introduction

Obesity or adiposity, which is rapidly increasing in both industrialized and non-industrialized countries, is a global epidemic that is constantly recognized as a health problem [1]. The World Health Organization estimated that among adults worldwide approximately 13% were obese and 39% were overweight in 2014. Obesity is defined as an imbalance between energy intake and energy expenditure [2]. Adipose tissues play an important role in the improvement of insulin resistance by regulating glucose and lipid metabolism as well as energy balance [3]. They also secrete a number of adipokines (e.g., chemokines, cytokines, and hormones) and produce free fatty acids (FFAs) that affect dyslipidemia [4]. Excessive fat accumulation leads to activation of the immune system and chronic low-grade inflammation. Increased circulating pro-inflammatory cytokines activate the immune system and affect insulin signaling and beta-cell dysfunction, which cause an insulin secretion disorder [5]. Dyslipidemia is characterized by elevated FFA, triglyceride (TG), total cholesterol (TC), and low-density lipoprotein-cholesterol (LDL-C) and decreased high-density lipoprotein-cholesterol (HDL-C) levels in plasma [6]. Obesity causes hypertension, cardiovascular disease, hyperlipidemia, insulin resistance, and type 2 diabetes mellitus [7]. It is also associated with non-alcoholic fatty liver disease (NAFLD). The prevalence and seriousness of this disease increase with the degree of obesity. NAFLD includes a spectrum of liver diseases that range from a fatty liver to steatohepatitis and cirrhosis without alcohol supplementation [8].

The artichoke (Cynara scolymus), which is an herbaceous plant belonging to the Asteraceae family, is usually grown in Mediterranean countries (e.g., Italy, Spain, France, and North Africa) and America. Traditionally, the artichoke has been used as a food and as an important remedy for promoting diuresis and a depurative for the care of rheumatism and gout. It still plays an important part in human nutrition especially in the Mediterranean region. Artichoke flower heads, which are commonly referred to as capitula, are immature composite inflorescences that are the edible part of the plant. Leaf extracts of the artichoke plant are used in herbal medicines as a hepato-protectant and choleretic and reportedly also have anti-carcinogenic, anti-oxidative, anti-human immunodeficiency virus, and antibacterial effects [9]. Artichoke leaves contain various phenolic compounds (about 7.13%) including chlorogenic acid (about 4.71%), cynarin (about 1.62%), luteolin (about 0.42%), cymaroside (about 0.25%), and 1-caffeoylquinic acid (about 0.13%) [10,11]. Among them, luteolin (LU), which is a member of the flavonoids (a subclass of flavones), exists in vegetables and fruits such as celery, parsley, and peppermint. It reportedly has antitumor, anti-inflammatory, and anti-obesity effects [12,13]. Liu J and colleagues [14,15] reported that luteolin ameliorates diet-induced obesity and insulin resistance in mice by activating AMPKα1 signaling in adipose tissue macrophages. However, there are few reports on the effects of the artichoke on obesity and related metabolic disorders. No studies on the effects of long-term supplementation with LU-enriched artichoke leaf (AR) are available.

Accordingly, the aims of the current study were to investigate the effects and possible underlying mechanisms of long-term supplementation with AR on diet-induced obesity (DIO) and related metabolic abnormalities (e.g., dyslipidemia, insulin resistance, and NAFLD) in C57BL/6N mice.

## 2. Materials and Methods 

### 2.1. Experimental Animals and Diets

Male C57BL/6N mice (four weeks old) were obtained from Orient Bio (Gyeonggi-do, Korea). All mice were individually housed under a constant temperature (24 °C) with a 12 h light/12 h dark cycle and fed a normal chow diet for 1 week after arrival. The mice were then randomly divided into four groups according to the following diets: normal diet (ND group, AIN-76 purified diet, *n* = 10), high-fat diet (HFD group, 20% fat, 1% cholesterol, *n* = 10), HFD with 0.005% (*w*/*w*) of LU (LU group, *n* = 8), and HFD with 0.005% (*w*/*w*) of AR (AR group, *n* = 10), fed for 16 weeks, respectively. The dried artichoke powder was incorporated directly into the HFD and the content of LU in the AR extract was 80% (*w*/*w*) (Shaanxi Jiahe Phytochem Co., Ltd., Xi’an, China). All experimental diets were prepared every week and stored in a dark room at −4 °C. At the end of the experimental period, all mice were anesthetized with ether after 12 h of fasting. Blood was taken from the inferior vena cava to determine the plasma lipid, adipokine, and hormone concentrations. The liver and adipose tissues were removed, rinsed with physiological saline, weighed, immediately frozen in liquid nitrogen, and stored at −70 °C until analysis. The animal study protocols were approved by the Ethics Committee at the Kyungpook National University (Approval No. KNU 2016-0040).

### 2.2. Morphology of the Liver and Fat Tissues

The liver and epididymal white adipose tissue (WAT) were removed from the mice, fixed in a buffer solution of 10% formalin, and embedded in paraffin for staining with hematoxylin and eosin. The stained areas were viewed under an optical microscope (Nikon, Tokyo, Japan) at a magnifying power of 200×.

### 2.3. Plasma Biomarkers

The plasma TC, HDL-C, and TG concentrations were determined with commercial kits (Asan Pharm Co., Seoul, Korea). The plasma FFA level was measured using an enzymatic kit (Wako Chemicals, Osaka, Japan). The apolipoprotein A-I (ApoA-I) and apolipoprotein B100 (ApoB100) levels were also determined using enzymatic kits (Eiken Chemical Co., Tokyo, Japan). The non HDL-C value, HDL-C to TC ratio (HTR), and atherogenic index (AI) were calculated using the following formulas: non HDL-C = [(TC) − (HDL-C) − (TG/5)], HTR (%) = (HDL-C)/(TC) × 100, and AI = [(TC) − (HDL-C)]/(HDL-C). The plasma insulin, the gastric inhibitory polypeptide (GIP), adipokine (leptin and resistin), and cytokine (interleukin 1beta (IL-1β), IL-6, and plasminogen activator inhibitor-1 (PAI-1)) levels were determined with a MILLIPLEX kit (Merck Millipore, Billerica, MA, USA). All samples were analyzed with a Luminex 200 Labmap system with XPONENT software (Luminex, Austin, TX, USA).

### 2.4. Fasting Blood Glucose, Intraperitoneal Glucose Tolerance Test, and Homeostatic Index of Insulin Resistance

After fasting the mice for 12 h, the blood glucose level was measured from the tail veins with a glucose analyzer, One Touch Ultra (Lifescan Inc., Wayne, PA, USA). The intraperitoneal glucose tolerance test (IPGTT) was conducted at week 15. In brief, after fasting the mice for 12 h, they were intraperitoneally injected with glucose (0.5 g/kg body weight). The blood glucose level was then measured from the tail vein at 0 min, 30 min, 60 min, and 120 min after the injection. The homeostasis model assessment (HOMA) was used to calculate the homeostatic index of insulin resistance (HOMA-IR) as follows: HOMA-IR = [fasting glucose (mmol/L) × fasting insulin (μIU/mL)]/22.5.

### 2.5. Hepatic and Fecal Lipid Contents

Hepatic and fecal lipids were extracted and dried [16]. Then the dried lipid residues were dissolved in 1 mL of ethanol for TG, cholesterol, and fatty acid (FA) assays. Triton X-100 and a sodium cholate solution in distilled water were added to 200 μL of the dissolved lipid solution to emulsify the molecules. The TG, cholesterol, and FA concentrations were measured using the same enzymatic kits used for the plasma analyses.

### 2.6. Preparation of Hepatic Subcellular Fractions

Hepatic mitochondrial, cytosolic, and microsomal fractions were prepared as previously described [17]. The mitochondrial fraction was used to measure the glucose-6-phosphatase (G6Pase) and carnitine palmitoyltransferase (CPT) activities while the cytosolic fraction was used to measure the malic enzyme (ME) and phosphoenolpyruvate carboxykinase (PEPCK) activities. The microsomal fraction was used to measure the phosphatidate phosphohydrolase (PAP) activity. The protein concentrations were measured using the bicinchoninic acid assay.

### 2.7. Lipid-Regulating and Glucose-Regulating Enzymatic Activities

ME activity was measured according to the method described by Ochoa et al. [18] and by monitoring the production of NADPH at 340 nm in the cytosol. PAP activity was measured using the method described by Walton and Possmayer [19]. CPT activity was determined using the method described by Markwell et al. [20]. PEPCK activity was measured in relation to the synthesis of oxaloacetate and the reduction of NADH to NAD by using the spectrophotometric assay developed by Bentle and Lardy [21]. G6Pase activity was determined using the method described by Alegre et al. [22] with slight modifications.

### 2.8. Analysis of Gene Expression

The liver and adipose tissues were homogenized in TRIzol reagent (Invitrogen, Grand Island, NY, USA) and the total RNA was reverse-transcribed into cDNA using the QuantiTect Reverse Transcription Kit (Qiagen GmbH, Hilden, Germany). The mRNA expression was quantified by real-time PCR (qPCR) using the SYBR Green PCR Kit (Qiagen) and SDS7000 sequence detection system (Applied Biosystems, Foster City, CA, USA). Gene specific mouse primers were used as suggested in Table 1. The amplification was performed as follows: 10 min at 90 °C, 15 s at 95 °C, and 60 s at 60 °C for a total of 35 cycles. The cycle threshold (Ct) was defined as the cycle at which a statistically significant increase in the SYBR Green emission intensity occurred. The Ct data were normalized using *GAPDH* and the relative gene expression level was calculated with the 2^∆∆*C*t^ method.

### 2.9. Statistical Analysis

The data are presented as the mean ± standard error of the mean. All statistical analyses were performed using SPSS (SPSS Inc., Chicago, IL, USA). Significant differences between the ND and HFD groups were determined using Student’s *t*-test and significant differences among the groups based on the HFD were determined by one-way analysis of variance (ANOVA). The results were considered statistically significant at *p* < 0.05. 

## 3. Results

### 3.1. Supplementation with AR and LU Lowered the Body Weight Gain and Adipose Tissue Weight by Regulating Lipid Metabolism-Related Adipocyte Gene Expression in Mice with DIO

**Figure 1:**
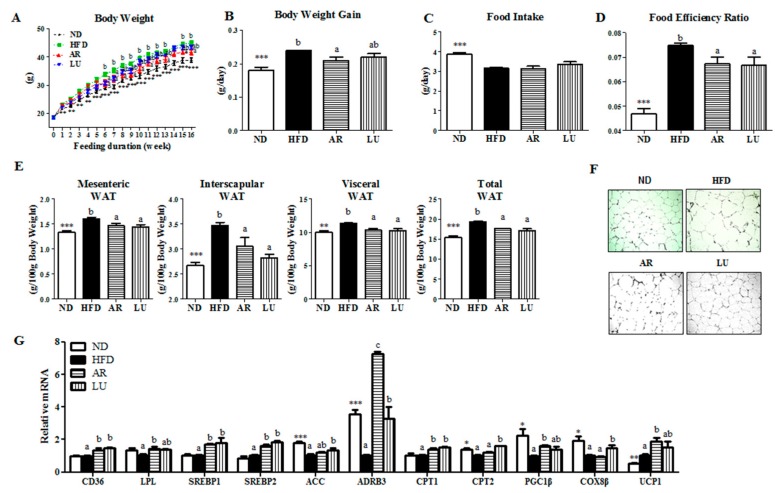
Effects of luteolin-enriched artichoke extract and luteolin on changes in the body weight (**A**), body weight gain per day (**B**), food intake (**C**), food efficiency ratio (**D**), white adipose tissue (WAT) weight (**E**), WAT morphology (magnification 200×) (**F**), and WAT gene expression (**G**) in C57BL/6N mice fed a high-fat diet (HFD). Data are the mean ± SEM. Significant differences between the HFD and ND groups are indicated: * *p* < 0.05, ** *p* < 0.01, *** *p* < 0.001. Bars marked with different lower case letters indicate significant differences among the groups based on the HFD (HFD, AR, and LU) at *p* < 0.05. Bars marked with the same lower case letters indicate no significant differences among the groups based on the HFD. ND, normal diet (AIN-76), HFD, high-fat diet (20% fat, 1% cholesterol), AR, HFD + 0.005% luteolin-enriched artichoke, LU, HFD + 0.005% luteolin. The food efficiency ratio is given as the body weight gain per food intake per day.

The body weight (BW) and body weight gain of the mice were significantly higher in the HFD group than in the ND group (Figure 1A,B). AR significantly decreased the BW after week 6 and consistently suppressed it except at week 14 (Figure 1A,B). In the HFD group, the food intake was significantly decreased while the food efficiency ratio (FER) was markedly increased when compared with the ND group (Figure 1C,D). Supplementation with AR or LU significantly decreased the FER relative to that in the HFD group (Figure 1D). The WAT weights were significantly higher in the HFD group than in the ND group. Both AR and LU significantly lowered the mesenteric, interscapular, visceral (sum of epididymal, perirenal, mesenteric, and retroperitoneal WAT) and total WAT (sum of visceral, subcutaneous, and interscapular WAT) weights in the HFD group (Figure 1D). Moreover, morphological observations revealed that the epididymal adipocyte size in the AR and LU groups was smaller than that in the HFD group (Figure 1F). 

The expression levels of lipid metabolism-related adipocyte genes were regulated by AR and LU supplementation. AR significantly increased not only the mRNA expression of adipocyte genes involved in both FA uptake and lipogenesis (i.e., *CD36*, *LPL*, *SREBP1*, and *SREBP2*) but also those related to FA oxidation (i.e., *ADRB3*, *CPT1*, *PGC1β*, and *UCP1*) relative to the levels in the HFD group (Figure 1G). LU also markedly elevated the adipocyte mRNA expression of both lipogenic genes (*CD36*, *SREBP1*, and *SREBP2*) and hydrolytic genes (*ADRB3*, *CPT1*, *CPT2*, and *COX8B*) relative to the levels in the HFD group (Figure 1G). Notably, AR significantly increased the expression of *ADRB3* relative to that in the LU group.

### 3.2. Supplementation with AR and LU Improved the Plasma Lipid Levels in Mice with DIO

The plasma TG, TC, HDL-C, non HDL-C, and AI levels were significantly higher while the HDL-C to TC ratio (HTR) and ApoA-I levels were significantly lower in the HFD group than in the ND group (Table 2). Supplementation with AR significantly decreased the plasma in FFA, TG, TC, and non HDL-C levels (Table 2). LU supplementation also significantly decreased the plasma FFA and tended to lower the plasma in TG, TC, and non HDL-C levels relative to those of the HFD group (Table 2).

### 3.3. Supplementation with AR and LU Lowered the Hepatic Lipid Levels by Modulating Hepatic Lipid-Regulating Enzyme Activities and Gene Expression and Increasing Fecal Lipid Levels in Mice with DIO

**Figure 2:**
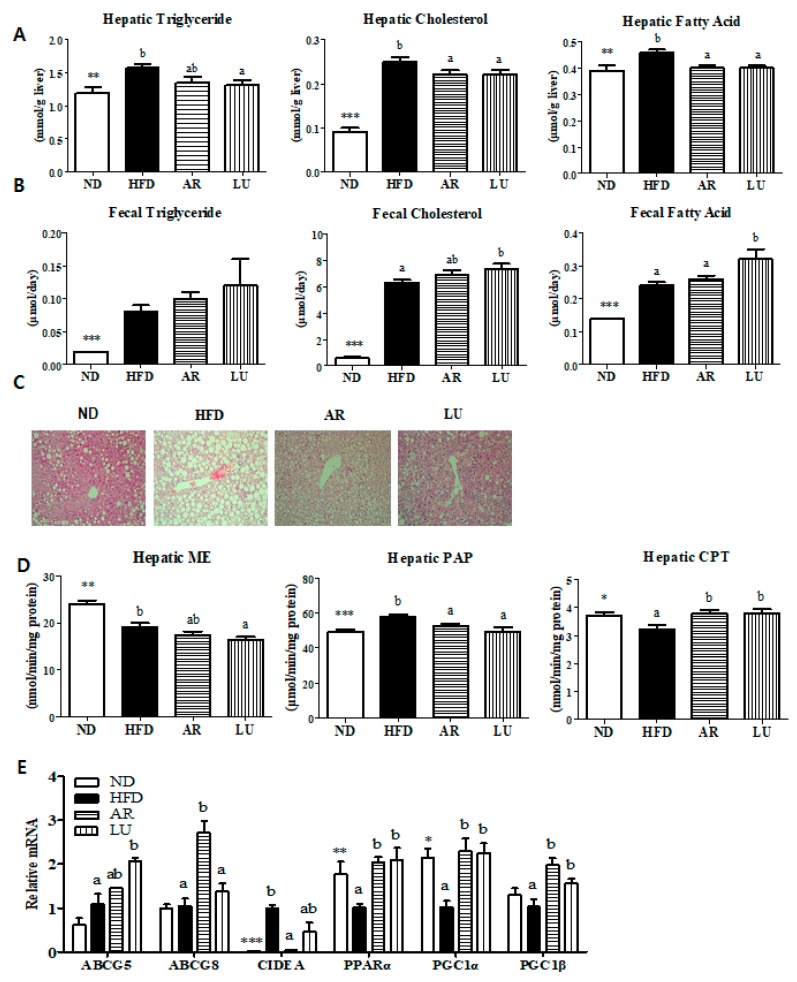
Effects of luteolin-enriched artichoke extract and luteolin on the hepatic lipid levels (**A**), fecal lipid levels (**B**), hepatic morphology (magnification 200×) (**C**), hepatic lipid-regulating enzyme activities (**D**), and hepatic gene expression (**E**) in C57BL/6N mice fed a high-fat diet. Data are the mean ± SEM. Significant differences between the HFD and ND groups are indicated: * *p* < 0.05, ** *p* < 0.01, *** *p* < 0.001. Bars marked with different lower case letters indicate significant differences among the groups based on the HFD (HFD, AR, and LU) at *p* < 0.05. Bars marked with the same lower case letters indicate no significant differences among the groups based on the HFD. ND, normal diet (AIN-76), HFD, high-fat diet (20% fat, 1% cholesterol), AR, HFD + 0.005% luteolin-enriched artichoke, LU, HFD + 0.005% luteolin, ME, malic enzyme, PAP, phosphatidate phosphohydrolase, CPT, carnitine palmitoyltransferase.

The hepatic and fecal TG, cholesterol, and FA levels were markedly higher in the HFD group than in the ND group (Figure 2A, B). Supplementation with LU significantly decreased the hepatic TG, cholesterol, and FA levels and increased the fecal cholesterol and FA contents relative to the levels in the HFD group (Figure 2A,B). AR significantly decreased the hepatic cholesterol and FA levels while it tended to increase the level of fecal cholesterol excretion relative to the levels in the HFD group (Figure 2A,B). Hepatic morphological observations revealed that the sizes and numbers of hepatic lipid droplets were significantly lower in the AR and LU groups than in the HFD group (Figure 2C). The AR and LU groups had significantly reduced hepatic PAP enzyme activity and increased hepatic CPT activity when compared with the enzyme activity levels in the HFD group (Figure 2D). Analysis of the hepatic gene expression levels showed that AR and LU supplementation increased the mRNA expression of *ABCG8* and *ABCG5* in the mice with DIO. These genes are involved in fecal cholesterol excretion, respectively (Figure 2E). In addition, AR decreased the expression of *CIDEA*, which is a gene involved in lipid accumulation. Both AR and LU significantly increased the mRNA expression of the fatty acid oxidation-related genes *PPARα*, *PGC1α*, and *PGC1β* relative to the levels in the HFD group (Figure 2E).

### 3.4. Supplementation with AR and LU Lowered Insulin Resistance and Glucose Tolerance by Modulating Hepatic Glucose-Regulating Enzymes in Mice with DIO

**Figure 3:**
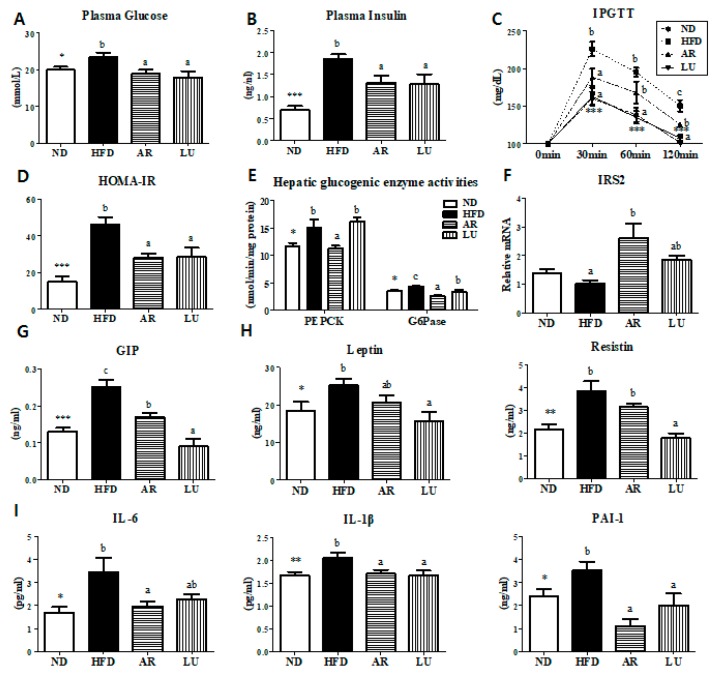
Effects of luteolin-enriched artichoke extract and luteolin on the plasma glucose (**A**) and insulin levels (**B**), IPGTT (**C**), HOMA-IR (**D**), hepatic glucogenic enzyme activity (**E**), hepatic *IRS2* gene expression (**F**), and plasma GIP (**G**), adipokine (**H**), and proinflammatory cytokine levels (**I**) in C57BL/6N mice fed a high-fat diet. Data are the mean ± SEM. Significant differences between the HFD and ND groups are indicated: * *p* < 0.05, ** *p* < 0.01, *** *p* < 0.001. Bars marked with different lower case letters indicate significant differences among the groups based on the HFD (HFD, AR, and LU) at *p* < 0.05. Bars marked with the same lower case letters indicate no significant differences among the groups based on the HFD. ND, normal diet (AIN-76), HFD, high-fat diet (20% fat, 1% cholesterol), AR, HFD + 0.005% luteolin-enriched artichoke, LU, HFD + 0.005% luteolin, IPGTT, intraperitoneal glucose tolerance test, HOMA-IR, homeostasis model assessment of insulin resistance, PEPCK, phosphoenolpyruvate carboxykinase, G6Pase, glucose-6-phosphatase, *IRS2*, insulin receptor substrate 2, GIP, gastric inhibitory polypeptide, IL, interleukin, and PAI-1, plasminogen activator inhibitor-1.

The IPGTT and HOMA-IR studies revealed that AR and LU supplementation had significantly reduced the plasma glucose and insulin levels as well as blood glucose levels relative to the levels in the HFD group (Figure 3A–D). Moreover, the activities of hepatic PEPCK and G6Pase were significantly suppressed while the expression of the hepatic insulin receptor substrate 2 (*IRS2*) gene was markedly increased by AR supplementation relative to the levels in the HFD group (Figure 3E,F). AR significantly reduced the plasma GIP, IL-6, IL-1β, and PAI-1 levels and LU markedly decreased the plasma GIP, leptin, resistin, IL-1β, and PAI-1 levels relative to the levels in the HFD group (Figure 3G–I).

## 4. Discussions and Conclusions

Obesity, which is defined as the accumulation of body fat, is associated with complications such as dyslipidemia, insulin resistance, type 2 diabetes, hypertension, and cardiovascular disease. In previous studies, the anti-oxidative effect of artichokes in modulating systemic oxidative stress was shown [23,24]. In addition, the artichoke was reported to decrease the plasma cholesterol level [25]. However, there are no reports of the effects of this plant on obesity and its associated complications. In the present study, we investigated the effects of an artichoke extract containing a high level of luteolin on adiposity, dyslipidemia, hyperglycemia, and inflammation through its modulation of lipid and glucose metabolism. 

We found that AR supplementation significantly decreased the body weight gain and fat mass by enhancing the expression of adipocyte fatty acid oxidation-related genes (*ADRB3*, *CPT1*, *PGC1β*, and *UCP1*), which inhibit the formation and accumulation of lipid droplets in adipose tissue. LU also markedly suppressed the fat mass and increased the adipocytes *ADRB3*, *CPT1*, *CPT2*, and *COX8B* gene expression levels. The CPT enzymes are considered to be involved in the main step of beta-oxidation and are involved in catalyzing the transfer of long-chain fatty acids from acyl-CoA to carnitine, which is then transported into the mitochondria [26]. CPT1 is located on the outer mitochondrial membrane while CPT2 is located on the inner mitochondrial membrane [27]. ADRB3 is known to play important roles in thermogenesis and lipolysis [28]. It stimulates lipolysis by increasing the cAMP concentration, which activates protein kinase A activity. Lipolysis generates FFAs at each step, which activate UCP1 directly [29]. UCP1 prevents the protons generated in oxidative phosphorylation from being used for ATP synthesis instead of directing them toward heat generation. Therefore, UCP1 regulates energy expenditure and is a major determinant of thermogenesis activity [30]. Expression of the FA-uptake genes (*CD36* and *LPL*) and lipogenic genes (*SREBP1*, *SREBP2*, and *ACC*) in adipose tissue was elevated by the AR and LU treatments. Adipose tissue protects against the accumulation of ectopic lipids in peripheral tissues such as liver and muscle by storing fat as a neutral lipid (triglyceride). The diversion of lipids from the muscle and liver into adipose tissue is generally beneficial for stimulating PPAR transcription factors, which have anti-inflammatory effects [13,31]. Together, these observations suggest that AR and LU not only activates lipogenesis in adipose tissue to prevent ectopic lipids in the liver but also simultaneously increases FA oxidation, which may contribute to reduced adiposity.

Obesity is associated with lipid metabolism abnormality [32]. The typical dyslipidemia of obesity consists of increased TG, FFA, TC, and LDL-C and decreased HDL-C [33]. In this study, supplementation with AR significantly decreased the plasma FFA, TG, TC, and non HDL-C levels. LU significantly decreased the plasma FFA level. In obesity, circulating FFAs can enter the liver. Enhanced levels of hepatic FFAs induce not only lipid synthesis and gluconeogenesis but also insulin resistance in the liver [4]. In the present study, AR supplementation noticeably improved the dyslipidemia, which was associated with the ameliorative effect of hepatic steatosis in mice with DIO. LU also markedly decreased the hepatic lipid levels and increased hepatic *ABCG5* mRNA expression and fecal TG and FA levels, which suggests that this flavonoid inhibits the hepatic lipid load by promoting biliary sterol excretion.

The alterations in hepatic lipid metabolism caused by AR and LU also revealed that their supplementation into an obesogenic diet for 16 weeks prevented hepatic steatosis by inhibiting lipogenesis and by increasing fatty acid oxidation in the liver of C57BL/6N mice. In the present study, the AR and LU treatments significantly decreased the hepatic PAP enzyme activity, which is related to TG synthesis and markedly increased the activity of hepatic CPT. This catalyzes the transfer of FAs from CoA to carnitine [34]. In addition, the AR and LU groups showed increased mRNA expression of fatty acid oxidation-related genes (*PPARα*, *PGC1α*, and *PGC1β*). The AR group also showed significantly decreased hepatic *CIDEA* mRNA expression. *CIDEA* expression is specifically induced by saturated FAs in which it induces the hepatic lipid droplet formation and TG accumulation events that cause hepatic steatosis [35]. 

The striking improvement of hepatic steatosis coupled with decreased adiposity in the AR-supplemented mice and LU-supplemented mice was associated with a normalization of the plasma glucose and insulin levels, which was a reflection of improved hepatic insulin sensitivity evidenced by the IPGTT and the reduced HOMA-IR. An excessive increase in glucose production causes fasting hyperglycemia, which leads to diabetes. Type 2 diabetes is characterized by an abnormal secretion of insulin that regulates glucose homeostasis [36]. Fasting hyperglycemia occurs as a result of the abnormal activities of hepatic glucose-regulating enzymes [37]. In this study, AR supplementation significantly decreased gluconeogenesis by suppressing hepatic PEPCK and G6Pase activities and increased hepatic *IRS2* mRNA expression. IRS knockdown has been shown to upregulate the lipogenic enzyme FAS and hepatic lipid accumulation [38]. 

Excess fat accumulation causes a low-grade inflammation that is related to metabolic diseases. Adipose tissue is an endocrine organ and secretes pro-inflammatory cytokines, which are implicated in obesity-related complications [39]. IL-6 is increased in obesity while IL-1β is a factor affecting TG accumulation and lipogenic enzyme activities [40,41]. IL-6 and IL-1β are pro-inflammatory cytokines that affect insulin resistance [42,43]. PAI-1, which causes cardiovascular diseases, is an increasingly important factor in obesity and the metabolic syndrome [44]. In the present study, AR significantly decreased the plasma pro-inflammatory cytokine (i.e., IL-6, IL-1β, and PAI-1) levels and LU also markedly reduced the plasma IL-1β and PAI-1 levels. Furthermore, AR and LU significantly decreased the plasma GIP levels. The gut-derived incretin hormone GIP, which is a 42-amino-acid polypeptide produced by the intestinal K cells, is secreted in response to the digestion of fat or glucose. GIP is one of the increasingly important factors in patients suffering from obesity or diabetes. It increases glucose-stimulated insulin secretion through specific receptors and regulates insulin secretion to maintain glucose homeostasis. In addition, GIP directly stimulates the release of pro-inflammatory cytokines from adipose tissue [45,46,47]. Therefore, these results suggest that the decreased level of plasma GIP by AR and LU is partially linked with glucose homeostasis and the decrease in inflammation, which leads to the prevention of obesity and consequently HFD-induced insulin resistance and hepatic steatosis.

**Figure 4:**
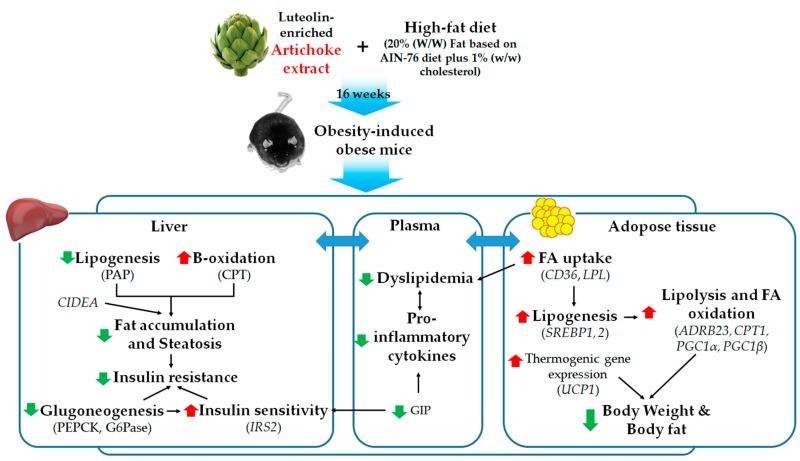
Proposed mechanisms for the anti-obesity effects of luteolin-enriched artichoke leaf (AR) extract. AR reduces adiposity by increasing adipocyte genes involved in lipolysis and FA oxidation as well as thermogenesis. In addition, AR not only activates FA uptake and lipogenesis in adipose tissue to prevent ectopic lipids in liver but also simultaneously increases FA oxidation, which may contribute to reduced adiposity, with improved dyslipidemia. AR also alters hepatic lipid and glucose metabolizing factors, which prevents hepatic steatosis by reducing the hepatic lipid load and eventually improving insulin resistance. This is associated with a decrease in the plasma pro-inflammatory cytokine levels.

In conclusion, this study demonstrated that AR supplementation improves adiposity and dyslipidemia by decreasing lipogenesis while increasing fatty acid oxidation, which contributes to the improvement of hepatic steatosis. LU also prevents adiposity and hepatic steatosis by decreasing lipogenesis while increasing biliary sterol excretion. In addition, both AR and LU supplementations prevent insulin resistance by decreasing gluconeogenesis and by improving inflammation. Although the anti-metabolic syndrome effects of AR and LU were similar in this study, the anti-adiposity and anti-dyslipidemic effects of AR were more evident than those of LU, which is a positive control. These results, therefore, suggest that there was a synergic effect between LU and other components such as chlorogenic acid in artichoke. Figure 4 illustrates the possible mechanisms behind the anti-obesity effects of AR and/or LU. Taken together, the results of this study suggest that 16 weeks of supplementation with the extract of artichoke containing a high LU concentration prevents obesity and metabolic disorders such as dyslipidemia, insulin resistance, and inflammation in mice with HFD-induced obesity.

## Figures and Tables

**Table 1:** Primer sequences used for RT-qPCR.

Gene	Primer Direction	Primer Sequence
Glyceraldehyde 3-phosphate dehydrogenase (*GAPDH*)	Forward	5′-CAAGTTCAACGGCACAGTCAAGG-3′
	Reverse	5′-ACATACTCAGCACCAGCATCACC-3′
ATP-binding cassette subfamily G member 5 (*ABCG5*)	Forward	5′-TCTTCCCTGAGCCTAGGGGG-3′
	Reverse	5′-CGATTAGCTCTTCCACCCGT-3′
*ABCG8*	Forward	5′-TTCACGCTCATAGTCGCTGGATAG-3′
	Reverse	5′-TGGTTCAATTCTCTTGGACACATCTTC-3′
Cell death-inducing DFFA-like effector A (*CIDEA*)	Forward	5′-TTTCAAACCATGACCGAAGTAGC-3′
	Reverse	5′-CCTCCAGCACCAGCGTAACC-3′
Peroxisome proliferator-activated receptor alpha (*PPARα*)	Forward	5′-CCTGAACATCGAGTGTCGAATAT-3′
	Reverse	5′-GGTCTTCTTCTGAATCTTGCAGCT-3′
PPAR-gamma coactivator 1alpha *(PGC1α)*	Forward	5′-AAGTGTGGAACTCTCTGGAACTG-3′
	Reverse	5′-GGGTTATCTTGGTTGGCTTTATG-3′
*PGC1β*	Forward	5′-GGTCCCTGGCTGACATTCAC-3′
	Reverse	5′-GGCACATCGAGGGCAGAG-3′
Sterol regulatory element-binding transcription factor 1a (*SREBP1a*)	Forward	5′-TAGTCCGAAGCCGGGTGGGCGCCGGCGCCAT-3′
	Reverse	5′-GATGTCGTTCAAAACCGCTGTGTGTCCAGTTC-3′
*SREBP2*	Forward	5′-CACAATATCATTGAAAAGCGCTACCGGTCC-3′
	Reverse	5′-TTTTTCTGATTGGCCAGCTTCAGCACCATG-3′
Acetyl-CoA carboxylase (*ACC*)	Forward	5′-GCCTCTTCCTGACAAACGAG-3′
	Reverse	5′-TGACTGCCGAAACATCTCTG-3′
Fatty acid synthase (*FAS*)	Forward	5′-GCTGCGGAAACTTCAGGAAAT-3′
	Reverse	5′-AGAGACGTGTCACTCCTGGACTT-3′
Lipoprotein lipase (*LPL*)	Forward	5′-GACTCGCTCTCAGATGCCCTAC-3′
	Reverse	5′-GCCTGGTTGTGTTGCTTGCC-3′
CD antigen 36 (*CD36*)	Forward	5′-TGGTGGATGGTTTCCTAGCCTTTC-3′
	Reverse	5′-TCGCCAACTCCCAGGTACAATC-3′
Adrenoreceptor beta 3 (*ADRB3*)	Forward	5′-ACCAACGTGTTCGTGACT-3′
	Reverse	5′-ACAGCTAGGTAGCGGTCC-3′
*CPT1*	Forward	5′-ATCTGGATGGCTATGGTCAAGGTC-3′
	Reverse	5′-GTGCTGTCATGCGTTGGAAGTC-3′
*CPT2*	Forward	5′-GCCTGCTGTTGCGTGACTG-3′
	Reverse	5′-TGGTGGGTACGATGCTGTGC-3′
Cytochrome *c* oxidase subunit 8B (*COX8B*)	Forward	5′-TGTGGGGATCTCAGCCATAGT-3′
	Reverse	5′-AGTGGGCTAAGACCCATCCTG-3′
Uncoupling protein 1 (*UCP1*)	Forward	5′-AGATCTTCTCAGCCGGAGTTT-3′
	Reverse	5′-CTGTACAGTTTCGGCAATCCT-3′

**Table 2:** Effects of luteolin-enriched artichoke extract and luteolin supplementation on the plasma lipid levels in C57BL/6N mice fed a high-fat diet.

	ND	HFD	AR	LU
**FFA (mmol/L)**	1.12 ± 0.01	1.16 ± 0.02 ^c^	0.98 ± 0.02 ^a^	1.07 ± 0.03 ^b^
**TG (mmol/L)**	0.94 ± 0.06 **	1.20 ± 0.05 ^b^	1.00 ± 0.04 ^a^	1.13 ± 0.02 ^ab^
**TC (mmol/L)**	6.28 ± 0.22 ***	8.70 ± 0.32 ^b^	6.90 ± 0.44 ^a^	8.07 ± 0.58 ^ab^
**HDL-C (mmol/L)**	1.64 ± 0.06 **	1.98 ± 0.09	1.81 ± 0.16	1.99 ± 0.11
**Non HDL-C (mmol/L)**	4.64 ± 0.17 ***	6.72 ± 0.29 ^b^	5.09 ± 0.30 ^a^	6.08 ± 0.51 ^ab^
**HTR**	26.20 ± 0.55 **	22.87 ± 0.93	26.08 ± 0.97	24.91 ± 1.39
**AI**	2.83 ± 0.08 *	3.41 ± 0.19	2.86 ± 0.14	3.06 ± 0.22
**ApoA-I (mg/dL)**	18.64 ± 0.22 *	17.74 ± 0.20 ^ab^	18.20 ± 0.16 ^b^	17.47 ± 0.29 ^a^
**ApoB100 (mg/dL)**	7.50 ± 0.55	10.12 ± 1.30	7.51 ± 0.97	8.15 ± 1.27
**ApoA-I/ApoB100**	2.57 ± 0.20	1.90 ± 0.20	2.76 ± 0.48	2.30 ± 0.34

Data are the mean ± SEM. Significant differences between the HFD and ND groups are indicated: * *p* < 0.05, ** *p* < 0.01, *** *p* < 0.001. There was a significant differences (*p* < 0.05) among the groups based on the HFD (HFD, AR, and LU) in the same line with different superscript letters (^a, b, c^). ND, normal diet (AIN-76), HFD, high-fat diet (20% fat, 1% cholesterol), AR, HFD + 0.005% luteolin-enriched artichoke, LU, HFD + 0.005% luteolin, non HDL-C = (TC) − (HDL-C), AI, atherogenic index = [(TC) − (HDL-C)]/(HDL-C); HTR = ratio of HDL-C to TC. TC, total cholesterol, HDL-C, high-density lipoprotein-cholesterol.

## Abbreviations

ABCG	ATP-binding cassette subfamily G member
ADRB3	adrenoreceptor beta 3
AI	atherogenic index
Apo	apolipoprotein
AR	artichoke
BW	body weight
CD36	CD antigen 36
CIDEA	cell death-inducing DFFA-like effector A
COX8B	cytochrome *c* oxidase subunit 8B
CPT	carnitine palmitoyltransferase
DIO	diet-induced obesity
FFA	free fatty acid
G6Pase	glucokinase, glucose-6-phosphatase
GIP	gastric inhibitory polypeptide
HFD	high-fat diet
HOMA-IR	homeostasis model assessment of insulin resistance
IL	interleukin
IPGTT	intraperitoneal glucose tolerance test
IRS2	insulin receptor substrate 2
LPL	lipoprotein lipase
LU	luteolin
ME	malic enzyme
ND	normal diet
PAI-1	plasminogen activator inhibitor-1
PAP	phosphatidate phosphohydrolase
PEPCK	phosphoenolpyruvate carboxykinase
PGC1	PPAR-gamma coactivator 1
PPAR	peroxisome proliferator-activated receptor
SREBP	sterol regulatory element-binding protein
UCP1	uncoupling protein 1
WAT	white adipose tissue
